# Two clusters of *Plasmodium knowlesi* cases in a malaria elimination area, Sabang Municipality, Aceh, Indonesia

**DOI:** 10.1186/s12936-018-2334-1

**Published:** 2018-05-02

**Authors:** Herdiana Herdiana, Irnawati Irnawati, Farah Novita Coutrier, Alfian Munthe, Mardiati Mardiati, Titik Yuniarti, Elvieda Sariwati, Maria Endang Sumiwi, Rintis Noviyanti, Paul Pronyk, William A. Hawley

**Affiliations:** 1 Child Survival and Development Cluster, UNICEF Indonesia Country Office, Jalan Sudirman Kav. 31, Wisma Metropolitan II, Fl 10th, Jakarta, 12920 Indonesia; 2 Paritrana Asia Foundation, Jakarta, Indonesia; 3 Municipal Health Authority of Sabang, Jalan By Pass Cot Ba’U, Sabang, Aceh Indonesia; 4 Eijkman Institute for Molecular Biology, Jalan Diponegoro, 69, Jakarta, 10430 Indonesia; 5 Ministry of Health, Sub Directorate of Malaria, Jakarta, Indonesia

**Keywords:** *Plasmodium knowlesi*, Aceh, Indonesia, Malaria elimination, Malaria diagnosis

## Abstract

In malaria elimination areas, malaria cases are sporadic and consist predominantly of imported cases. *Plasmodium knowlesi* cases have been reported throughout Southeast Asia where long-tailed and pig-tailed macaques and *Anopheles leucosphyrus* group mosquitoes are sympatric. The limitation of microscopic examination to diagnose *P. knowlesi* is well known. In consequence, no *P. knowlesi* case has previously been reported from routine health facility-based case finding activities in Indonesia. This report describes two clusters of unexpected locally acquired *P. knowlesi* cases found in an area where *Plasmodium falciparum* and *Plasmodium vivax* infection had been eliminated in Sabang Municipality, Aceh, Indonesia. The difficulties in diagnosis and response illustrate challenges that Southeast Asian countries will increasingly face as the formerly common malaria parasites *P. falciparum* and *P. vivax* are gradually eliminated from the region.

## Background

As a result of universal coverage of long-lasting insecticide-treated bed nets (LLINs), indoor residual house spraying (IRS), early diagnosis by microscopy, and radical treatment using artemisinin combination therapy (ACT) and primaquine (PQ), the number of malaria cases has markedly declined throughout Indonesia [1]. Sabang municipality in far western Indonesia reduced malaria incidence from 100.9 per 1000 population in 2001 to 0.13 in 2011 [2], to zero since then, with the last locally transmitted case reported in a child in 2011.

Building upon this accomplishment, the municipality reoriented its programme to prevent local transmission subsequent to parasite importation, which occurs sporadically [3]. Surveillance should detect symptomatic and asymptomatic malaria cases, identify their origin, and prevent onward local transmission from imported cases. Following World Health Organization (WHO) recommendations, Sabang applied active case detection (ACD), case investigation (CI), reactive case detection (RACD), treatment follow-up at days 3, 7, 14, 21, 28, and 90 (for *Plasmodium vivax*), and directly observed treatment (DOT); in addition, a migration surveillance system was developed. ACD is conducted by village malaria volunteers known in Indonesia as a *Juru Malaria Lingkungan* (JML). JMLs survey the neighbourhood for fever cases regularly, take blood smears, and send them to the health centre for analysis. JMLs also assist health staff with case investigations when a positive diagnosis is obtained. CI is followed by RACD within 1–7 days to detect additional cases in the neighbourhood of the index case. Finally, since populations at risk are well known, people may be screened by JMLs after returning to Sabang from a malaria-endemic area.

From 2011 to 2013, the surveillance system detected 12 imported cases (6 *P. vivax* cases, 4 *Plasmodium falciparum* infections and 2 mixed infections of *P. falciparum* and *P. vivax*) with no subsequent local transmission. In 2014, the system unexpectedly detected two clusters of indigenous malaria via PCD and community-based ACD. In all, of 18 cases were detected in six villages, 11 cases were detected in 2 clusters encompassing 2 villages. Although the cases were originally identified via microscopy as *Plasmodium malariae* and *P. vivax*, case investigation raised suspicion that *Plasmodium knowlesi* might be involved, leading to subsequent PCR diagnosis and confirmation that 15 of the cases were *P. knowlesi* infections, with two patients declining to provide blood for diagnosis, and one negative. Of the 15 PCR-confirmed *P. knowlesi* cases, all were treated, with 11 negative by microscopy upon follow-up through day 28, two patients negative upon incomplete follow up through day 21, and one case negative through day 14, while two patients were lost to follow up. This report describes individual case histories and response of the local health system to this outbreak in the hope that it can inform programmatic efforts elsewhere. It also discusses the possibility that non-zoonotic transmission may have occurred.

Though cases where found throughout the island, we define two clusters as having occurred, due to close proximity in space and time. One cluster occurred in the northwest part of the island, in Iboih village, consisting of 8 cases (case numbers 7–14) within a 24-day period. The second cluster of three cases (numbers 15–17) occurred in one family within a week in the north-eastern part of the island.

**Fig. 1:**
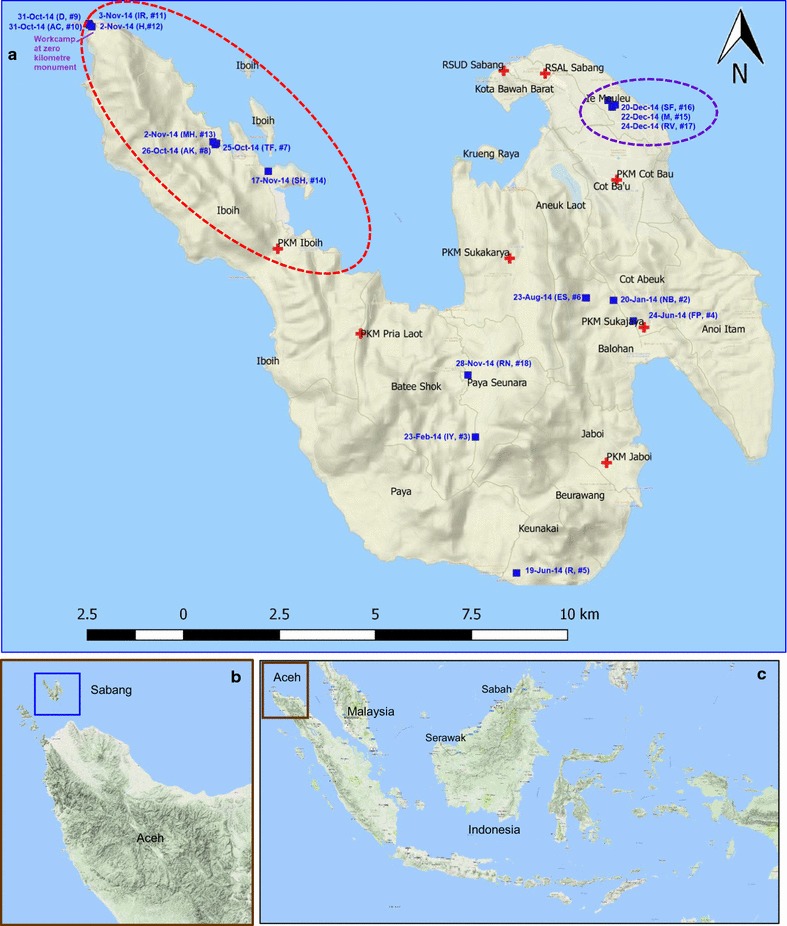
**a** Distribution of *Plasmodium knowlesi* cases based on estimated date of first presentation of symptoms. Initials and case number of patients correspond with those in Table 1. 15 PCR confirmed Pk cases and two suspected Pk cases (SF and RV from Table 1) are mapped. Red cross: position of health facilities in Sabang including both Primary Health (PHC) and hospitals (RSUD and RSAL). Dotted red ellipse: cluster of cases at Iboih Village. Dotted purple ellipse: cluster of cases in a family at Ie Meulee Village. **b** Position of Sabang within Aceh Province. **c** Position of Sabang within the Indonesian archipelago and Malaysia

**Fig. 2:**
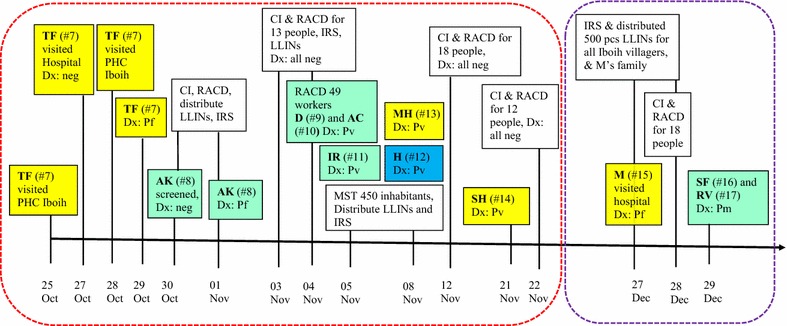
Timeline of events for two clusters of *Plasmodium knowlesi* cases in Sabang. For all cases case investigations and reactive case detection were performed; followed by IRS and LLIN distribution to index case’s house. On 27–29 December 2014, IRS and mass LLIN distribution was done for all residents of Iboih village. Yellow box: passive case detection; green box: reactive case detection; blue box: mass screening and treatment; white box: response of Municipal Health Office. Bold letters: initial of patient. Hashtag number in bracket: case ID number. Dotted red rectangle: a cluster at Iboih Village. Dotted purple rectangle: a family cluster at Ie Meulee Village. Dx: diagnosis, PHC: Primary Health, CI: case investigation, RACD: reactive case detection, MST: mass screening and treatment, LLINs: Long-lasting insecticide-treated nets, IRS: indoor residual spraying

Figure 1 shows the distribution of cases in Sabang and the dates on which symptoms are presumed to have commenced. A detailed narrative for each case illustrates the methods by which cases were detected and are summarized in Table 1, while Fig. 2 provides a detailed timeline for cases in the two clusters.

**Table 1:** Result of diagnosis of suspected malaria cases in Sabang at Primary Health Centres (PHC), Municipal Health Office (MHO), Provincial Health Office (PHO), and Eijkman Institute of Molecular Biology (EIMB) in 2014

Case ID	Initial of patient	Age	Fever duration (days)	Clinical data	Village	Case finding method	Date of first symptoms presented
Non-cluster							
1	S	28	15	Fever, chill, nausea, vomiting, headache	Kuta Timur	PCD	20 Dec 2013
2	NB	27	7	Fever	Balohan	PCD	20 Jan 2014
3	IY	27	7	Fever, chill, vomiting, headache, nausea, unconsciousness^a^, haemoglobinuria	Batee Shok	PCD	22 Feb 2014
4	FP	21	7	Fever, chill	Balohan	ACD	24 Jun 2014
5	R	17	13	Fever, chill, nausea	Keuneukei	ACD	19 Jun 2014
6	ES	30	7	Fever, headache, nausea	Balohan	PCD	26 Aug 2014
18	RN	12	5	Headache, Haemoglobinuria	Batee Shok	PCD	28 Dec 2014
Cluster 1 at Iboih Village							
7	TF	70	7	Unconsciousness, muscle weakness, haemoglobinuria, feet oedema, no appetite, chill, nausea, hypotension,	Iboih	PCD	25 Oct 2014
8	AK	45	7	Unconsciousness, severe headache	Iboih	PCD	26 Oct 2014
9	D	27	5	Fever, chill, headache	Iboih	RACD	31 Oct 2014
10	AC	26	5	Fever, chill, headache, nausea, cough, abdominal pain	Iboih	RACD	31 Oct 2014
11	IR	18	3	Fever, chill	Iboih	RACD	3 Nov 2014
12	H	21	7	Fever, chill, nausea	Iboih	MST	2 Nov 2014
13	MH	6	7	Fever	Iboih	RACD	2 Nov 2014
14	SH	22	5	Fever, chill, headache	Iboih	PCD	17 Nov 2014
Cluster 2 at Ie Meulee Village							
15	M	16	6	Fever, chill	Ie Meulee	PCD	22 Dec 2014
16	SF	38	10	Fever	Ie Meulee	RACD	20 Dec 2014
17	RV	12	3	Fever	Ie Meulee	RACD	24 Dec 2014

Case ID	Date of first diagnosed as malaria	Microscope result at PHC	Microscope result at MHO	Microscope result at PHO	Parasite density (per µL)	PCR result at EIMB	Classification
Non-cluster							
1	2 Jan 2014	Pv	Pv	NA	2680	Neg	Imported
2	27 Jan 2014	Pv	Pv	NA	2085	Pk	Imported
3	28 Feb 2014	Pm	Pm	Pm	14,620	Pk	Indigenous
4	3 Jul 2014	Pm	Pm	NA	2480	Pk	Indigenous
5	1 Jul 2014	Pf	Pm	Pm	4800	Pk	Indigenous
6	1 Sep 2014	Pv	Pm	Pm	3450	Pk	Indigenous
18	1 Jan 2015	Pm	Pm	NA	2300	Pk	Indigenous
Cluster 1 at Iboih Village							
7	28 Oct 2014	Pf	Pv, Pm, Mixed (Pv + Pm)	Mixed (Pf + Pv)	24,368	Pk	Indigenous
8	3 Nov 2014	NA	Pv	Pf	48	Pk	Indigenous
9	4 Nov 2014	NA	Indetermine	Pv	1300	Pk	Indigenous
10	4 Nov 2014	NA	Pv	Pv	2140	Pk	Indigenous
11	5 Nov 2014	NA	Pv	Pv	840	Pk	Indigenous
12	8 Nov 2014	NA	Pv	Pv	1240	Pk	Indigenous
13	8 Nov 2014	NA	Pv	NA	2450	Pk	Indigenous
14	21 Nov 2014	Pv	Pm	NA	2130	Pk	Indigenous
Cluster 2 at Ie Meulee Village							
15	27 Dec 2014	Pf	Pm	NA	1986	Pk	Indigenous
16	29 Dec 2014	NA	Pm	NA	884	NA	Indigenous^b^
17	29 Dec 2014	NA	Pm	NA	426	NA	Indigenous^b^

^a^Unconsciousness defined as GCS 12–13

^b^Suspected *P. knowlesi* case

## Site

Sabang Municipality is part of Aceh Province, and is located at the north-western tip of Indonesia (Fig. 1). The municipality has a land area of 153 km^2^ [4] of which 97.43 km^2^ is forest [5]. The highest altitude is Jaboi mountain in Sukajaya sub-district at 308 masl. This municipality consists of five islands: Weh, Klah, Rubiah, Seulako, and Rondo. Only the largest island, Weh, has permanent residents totaling 33,622 people. Administratively, Sabang Municipal is divided into two subdistricts, namely Sukakarya and Sukajaya, and 18 villages [4].

## Malaria transmission

Prior to elimination, malaria transmission occurred every month in Sabang, with a peak during the rainy season from November to January [2]. Malaria cases have been reported from all villages, and a vector survey in 2008 found *Anopheles sp.* in 15 villages. Larvae of *Anopheles aconitus*, *Anopheles dirus*, *Anopheles sundaicus*, *Anopheles. subpictus*, *Anopheles montanus*, *Anopheles barbirostris*, *Anopheles kochi*, *Anopheles umbrosus*, *Anopheles hyrcanus*, and *Anopheles vagus* were found, while night time human bait collections found *An. aconitus*, *An. dirus, Anopheles flavirostris*, *An. sundaicus*, *An. subpictus* and *Anopheles minimus* [2].

Sixty percent of Sabang is forested, with long-tailed macaques present in both the forested interior and in the forest fringe. A recent study found between 10 and 25 *M. fascicularis* present in a protected forest near Iboih village [6].

## Case narratives

### Non-cluster cases

*Case 1*. A 28-year old man (S) visited Primary Health Centre (PHC) Sukakarya on 3 January, 2014 with fever, chill, nausea, vomiting, and headache for 2 weeks after he arrived in Sabang from Bireun District, Aceh, which is not known to be endemic for malaria [7]. He arrived Sabang during the third week of December 2013, where he worked at a construction site in Kuta Timur village, Sabang. Diagnosis from the PHC and the Municipal Health Office (MHO) microscopist was *P. vivax* with treatment by 3 days dihydroartemisinin piperaquine (DHP) + 14 days PQ. Follow-up diagnosis for days 3 and 7 were negative with no further follow-up as he returned to his hometown outside Sabang. One week prior to presentation at the PHC, PHC staff and a JML performed migration surveillance at his camp site, but he refused to be screened. Eighteen co-workers were screened but no positive malaria cases were detected. PHC and MHO staff classified S’s case as imported.

*Case 2*. NB, a 27-year old man, and boat crew member arrived Sabang from Aceh Besar district in northern Sumatra. He had consistently stayed overnight in Aceh Besar, but came back to Sabang when he came down with fever as his family lives in Sabang. He had a history of fever of 7 days when presented to PHC Sukajaya on 27 January, 2014. He was diagnosed with *P. vivax* and treated for 3 days with DHP + 14 days PQ. Follow-up was conducted on days 3, 7 and 14 with negative blood smear tests for every visit. RACD was performed for 8 neighbours with negative results. This case was likely imported.

*Case 3*. IY, a 27-year old man and driver with frequent travel between Sabang and the Aceh mainland (there is routine ferry service between the two). On 28 February, 2014, he sought treatment at PHC Sukakarya with symptoms of 7 days of fever, chills, vomiting, headache, nausea, unconsciousness with a Glasgow Coma Score of 12 and blackwater urine. The PHC, MHO Sabang and provincial microscopists all reported his blood smear test positive for *Plasmodium malariae*. He was treated with DHP + PQ, with DOT conducted by a nurse. Follow-up was done on days 3, 14, 21, and 28 with no parasites found on his blood smear. He had stayed overnight at his workplace campsite at Batee Shok Village in Sabang for 1 month prior to emergence of symptoms. MHO Sabang classified his case as indigenous. PHC staff performed RACD at the workplace campsite, with 27 people screened by microscopy; all were found negative for malaria infection.

*Case 4*. FP, a 21-year old business man and farmer had a week of fever and was screened by a JML on 3 July 2014 via ACD. According to thick and thin blood smears sent to PHC Sukajaya by JML, the result was *P. malariae*, which was subsequently confirmed by the district microscopist. The case was treated with DHP + PQ, with treatment observed by the JML. Follow-up screening was complete with results all negative. Although he had travelled outside Sabang to a non-endemic district before diagnosis, CI revealed that he had been febrile before travel and during his travel. He frequently visited his farm at forest fringe. RACD was conducted near his residence on 20 people, with all negative.

*Case 5*. On 1 July, 2014, PHC Jaboi reported one *P. falciparum* microscopy-diagnosed case found through ACD. R, a 17-year old student, had 13 days of fever, chill and nausea but had not sought treatment from any health provider until he was interviewed by a JML. Both the MHO and provincial microscopist diagnosed him with *P. malariae* infection. The case was treated by DHP + PQ, DOT was performed by JML, and blood smear tests on days 7, 14, 21, 28, and 90 were negative. He had no travel history outside Sabang. His house was located within 500 m of the forest and 500 m from a stream. Twenty-one neighbours were screened via RACD, with negative results.

*Case 6*. A 30-year old farmer, ES, had visited PHC Sukajaya complaining of nausea, a week of fever, and headache. He was diagnosed by PHC Sukajaya with *P. vivax* on 1 September, 2014; MHO and provincial microscopist concluded that he was infected with *P. malariae*. He received DHP + PQ with DOT performed by a JML. He recovered, with blood smears negative from days 3 to 90. No travel history outside Sabang was reported, but his house was located near the forest and he frequently visited his farm near the forest. Twenty of his family members and neighbours were screened and no other secondary cases were detected.

*Case 18*. At 28 December, 2014, RN, a 12-year old female student from Batee Shok village visited PHC Pria Laot with 5 days of fever, which a general practitioner had diagnosed as common cold, thus no malaria test was done. Because of lack of improvement, RN visited the district hospital with headache and haemoglobinuria on 1 January, 2015 where the microscopist diagnosed her as infected with *P. falciparum*. On the same day, MHO Sabang investigated the case and family, and cross-checked RN’s blood smear. The MHO microscopist decided the case was *P. malariae*, but suspected that it might be *P. knowlesi.* The case was treated with DHP + PQ with complete follow-up on day 3, 7, 14, 21, and 28. All follow-up smears were negative. In addition, 30 family members and neighbours were screened with negative results. On 2 January, 2015, the MHO Sabang responded with house spraying within a radius of 1 km from the index case, with LLINs also distributed to RN’s family members. RN’s house was located in the forest fringe, where macaques were observed.

### Cluster 1 at Iboih Village

*Case 7*. TF, a 70-year old man and hostel owner presented with fever at PHC Iboih on 25 October, 2014, but his blood smear was read as negative. On 27 October 2014, still suffering from fever, chills and nausea, TF sought treatment at the district hospital; his blood smear was negative. The patient returned to PHC Iboih on 28 October, 2014, complaining of 7 days of fever, chills, nausea, no appetite, muscle weakness, unconsciousness, and feet oedema. His blood pressure was 90/70 mmHg, pulse rate 90/min, respiration rate 24/min, axilla temperature 37.2 °C. Another thick and thin blood smear was taken and read as positive for *P. falciparum*. PHC staff sent a case notification to MHO Sabang the same day. The blood smear was cross-checked by 4 certified microscopists at the MHO on 30 October, 2014, but interpretation varied as to whether the parasite was *P. vivax, P. malariae,* or a mixed infection of *P. vivax* and *P. malariae*. Parasite density was 24,368/µl blood. The blood smear was then sent for examination at the Provincial Health Office by the certified microscopist, who interpreted the infection as a mixture of *P. falciparum* and *P. vivax*. The patient was treated with DHP for 3 days and PQ at 0.75 mg/body weight for day 1, and 0.25 mg/body weight for days 2–14. Follow-up blood smears were negative for days 3, 7, 14, 21, 28, and 90. In response to the positive diagnosis on 28 October, the PHC Iboih and MHO carried out CI and RACD on 29 and 30 October, 2014. The subject had no travel history to a malaria-endemic area; the case was classified as indigenous. Thirteen residents residing within a radius of 500 m from the TF’s house were screened and found negative. TF lives in a hilly, forested area and *Anopheles* larvae were found near his house. On 1 November, 2014, MHO Sabang distributed LLINs and sprayed his house and his 7 nearest neighbours’ houses.

*Case 8*. On 1 November, 2016, AK, a -ear old man and a shop owner visited PHC Iboih complaining of a week of fever and a severe headache. He had sought treatment from a private nurse 1 week previously. AK was screened on 30 October, 2014 during the RACD of TF, but his blood smear was negative. Nonetheless, because of his complaints of severe headache and fever, venous blood was taken and sent to the MHO Sabang. Microscopists there found low density *P. vivax* with only 3 parasites detected from all fields. The diagnosis was cross-checked by a provincial microscopist who determined that the parasite was *P. falciparum*. The patient received treatment of 3 days DHP and 14 days PQ as per mixed infection protocol. The patient was hospitalized for 3 days, and follow-up blood smears on days 3, 7, 14, 21, 28, and 90 were all negative. Case investigation was carried out on 3 November, 2014. The investigation determined that the patient owned a tourist shop in the farthest western point of Sabang and frequently spent the night there. AK’s house was located about 500 m from TF, and less than 1 km from the forest. His shop is in the forest and near a road construction site. Because AK split his time between his residence and his work site, the MHO Sabang carried out RACD at both locations. On 3 November, his 5 family members and 7 neighbours were screened and found negative. Work site screening covered only individuals with fever or a history of fever; 49 construction workers were screened, constituting about 80% of those living in the area. This screening yielded 3 microscopy-positive male workers: D, 27-year old; AC, 26-year old, and IR, 18-year old. D and AC were diagnosed on 4 November, 2014, while IR was diagnosed on 5 November, 2014.

*Case 9*. D reported fever for 5 days, headache and chills. His blood pressure was 100/70 mmHg, and axilla temperature 38 °C. His initial blood smear could not be identified to species by the MHO microscopist; it was sent to the provincial laboratory where a diagnosis of *P. vivax* was made. The patient was hospitalized for 3 days in PHC Iboih and treated with 3 days’ DHP + 14 days of PQ as AK doses. Follow-up was incomplete as he returned to his home town outside of Sabang on day 4 after initial treatment; however, on day 4 his blood smear was negative.

*Case 10*. AC reported 5 days of fever, chills, headache, nausea, cough, and gastritis. His blood pressure was 160/70 mmHg, pulse rate 72/min, respiratory rate 28/min and axilla temperature 39 °C. His blood smear was positive for *P. vivax*, as determined by both the MHO and provincial microscopists. AC was admitted to PHC Iboih on 4 November, 2014 and treated with 3 days’ DHP + PQ for 14 days. DHP consumption was directly observed. Follow-up was done on day 3 upon discharge; his smear was negative but no additional follow-up was done as he returned to his home town outside of Sabang.

*Case 11*. IR was admitted to PHC Iboih on 5 November, 2014 with a history of 3 days of fever and chills. His blood pressure was 100/70 mmHg, pulse rate 68/min, respiratory rate 18/min and axilla temperature 38 °C. Both the MHO and provincial microscopists diagnosed him as suffering from infection with *P. vivax*. He was treated for 3 days with DHP + 14 days PQ for mixed infection doses. He was discharged after 1 day but follow-up smears were taken on days 3, 7, 14, and 21. All were negative.

The workers had been in residence at the construction site near the zero (0) kilometre monument (which marks the westernmost point of Indonesia at Fig. 1) for about 1 month prior to detection of the first infection. They slept under a temporary wood shelter with no mosquito net. The environment is forested and both long-tailed and pig-tailed macaques are common.

In response to the cases detected within a week in Iboih village, MHO Sabang expanded RACD to all individuals residing within 500 m of an index case house. In addition, all construction workers were screened. Mass screening and treatment (MST) was conducted at Iboih village aimed to prevent further transmission. However, all 450-people screened were negative. The MHO also distributed LLINs to all villagers and sprayed houses in the village as well as at the construction workers’ camp.

*Cases 12 and 13*. Concurrent with the MST, a construction worker (H, a 21-year old man, case 12) and a primary school student (MH, a 6-year old boy, case 13) sought treatment at PHC Iboih on 8 November, 2014. H complained of 7 days of fever, chills and nausea. His blood pressure was 120/80 mmHg, pulse rate 72/min, respiratory rate 20/min and axilla temperature 36.5 °C. Both the MHO and provincial microscopist diagnosed the case as *P. vivax.* He was treated according to mixed infection protocol for 3 days with DHP + 14 days of PQ. Follow-up on days 3, 7, 14, and 21 was negative. MH presented with 7 days of fever. On 8 November, the MHO microscopist diagnosed him as positive with *P. vivax*. Although he lived near AK, he was not screened during the RACD on 3–4 November because he was outside of Sabang (Bireun), a non-malaria endemic area, to visit his grandfather for 1 week. Other that this visit, he had no travel history except to the primary school near his house. He was treated for 3 days DHP, plus PQ for 14 days, with treatment observed by his mother. His smears were negative on days 3, 7 and 14, when he was lost to follow-up as his family travelled outside of Sabang. On 12 November, 18 people living near MH were screened, all were negative.

*Case 14*. On 21 November, 2014, SH, a 22-year old man and hostel receptionist, visited PHC Iboih with complaint of 5 days of fever, chills and headache. The PHC microscopist diagnosed the case as *P. vivax*, while the district microscopist diagnosed the smear as *P. malariae*. SH has lived in Sabang since 2010, but frequently travels to his home town in non-endemic Bireun District. He was treated with DHP and PQ with negative follow-up on days 3, 7, 14, 21, and 28. RACD was performed for his 12 closest neighbours, all negative.

### Cluster 2 at Ie Meulee Village

*Case 15*. On 27 December, 2014, M, a 16-year old male student from Ie Meulee village visited Sabang Municipal Hospital with a 6-day history of fever and chills. The hospital microscopist diagnosed him with *P. falciparum*, while the MHO microscopist identified the species as *P. malariae*. RACD was conducted near M’s house with 18 people screened on 28 December, 2014. Two additional cases were found, both family members: SF (case 16), a 38 year old woman (M’s mother) and RV (case 17), a 12 year old student (M’s sister). Both additional cases were diagnosed as *P. malariae* with the appearance of parasites in the blood smear similar to M’s smear. Case investigation revealed that SF had history of intermittent fever in the last 10 days, while RV presented with intermittent fever 3 days after M. The 3 cases were treated by 3 days DHP and 14 day PQ. M and SF were hospitalized for 3 days at the Municipal Hospital. All blood smear test results during treatment follow-up on days 3, 7, 14, 21, 28, and 90 were negative. This family’s house is located fewer than 500 m from the forest fringe, and macaques were observed in the yard surrounding the house.

The MHO periodically followed up all cases in Sabang through December 2015. No complications or relapses were detected. Surveillance activities continued, including ACD by JML, migration surveillance, and RACD, with special attention paid to the zero kilometre monument and associated construction campsite. These activities detected three additional microscopy-diagnosed *P. malariae* cases: in July 2015 at Keunekei Village, in September 2015 at Iboih Village, and in September 2016 in Ie Meulee Village (but likely infected at Iboih Village). RACD following these three cases yielded no additional positive cases.

### PCR confirmation

Because of the varying interpretations of the blood smears taken during this outbreak, venous whole blood from all cases was sent to the Eijkman Institute in Jakarta for species-specific PCR examination. As explained elsewhere [8, 9], the methodology for PCR examination was by nested PCR using the cytochrome b gene and AluI enzyme digestion, then followed by 18S rDNA nested PCR. Aceh Province surveillance guidelines recommend molecular identification of malaria cases in the provinces [10], but no specific funding for this was allocated in Sabang Municipality’s work plan. Of the 16 samples PCR tested in 2014, Eijkman Institute reported 15 *P. knowlesi* infections and one negative (Table 1).

## Conclusion

The two clusters of *P. knowlesi* reported here were surprising to local authorities at the time of the outbreak, as the last reported case of locally transmitted malaria on the island was recorded in 2011. *Plasmodium knowlesi* was first formally reported in Aceh in 2016 [9], with the reported detection of this parasite on the main island of Sumatra in 2014. In retrospect, the occurrence of *P. knowlesi* on Sabang is not surprising, given the presence of *Anopheles leucosphyrus* group mosquitoes [2, 11] and the common occurrence of macaques in the municipality. This is consistent with modelling that has predicted the geographical distribution of *P. knowlesi* based upon sympatry of *An. leucosphyrus* group mosquitoes and macaques [11].

One cluster of cases was associated with a construction site on the far western tip of Sabang. Workers at this site had a history of staying overnight in the forest, without protection from mosquitoes, in an area where macaques are common. All of the cases were males and had the same forest exposure risk, with characteristics consistent with *P. knowlesi* cases from other areas [9, 12, 13]. In contrast, the second cluster occurred in a residential location near the forest and consisted of three people from one family (mother and two teenagers), suggesting that transmission probably occurred inside or close to the houses. This finding is consistent with a recent report from Sabah [14]. This second cluster is consistent with either zoonotic or human to mosquito to human transmission of the parasite, but it is not possible to make a definitive conclusion.

Even though Sabang had transitioned to maintenance phase of malaria elimination, the outbreak of *P. knowlesi* highlighted the need for continued intense surveillance. The measures taken by the MHO via ACD, RACD and vector control apparently put an end to outbreak, but it is noteworthy that sporadic cases of *P. knowlesi* continue to occur in Sabang, with 18 PCR-confirmed cases from 8 scattered villages occurring in 2017.

This report emphasizes the difficulty of maintaining high-quality microscopy in a public health setting as has been observed from other studies where multiple species of *Plasmodium* are co-endemic [14, 15]. Misdiagnosis of *P. knowlesi* as *P. falciparum, P. vivax* or *P. malariae* has been reported in Sabah [14], Sarawak [15], and Aceh Besar [9]. In Sabang, local microscopists had not been trained to identify *P. knowlesi* from blood smear slides, so it was unsurprising that none identified the parasite correctly. In Indonesia, *P. knowlesi* cases had only been reported from Kalimantan (Borneo island) [16, 17] and just recently detected at Aceh Besar [9], Batubara, Langkat and South Nias [18] in Sumatra. Thus, Sabang’s microscopists were not familiar the appearance of this parasite.

As a consequence of inaccuracy of microscopic identification, treatment administered to a patient may be inadequate. Indonesia’s national malaria treatment guidelines [19] do not yet cover *P. knowlesi*. While treatment for *P. falciparum* is 3 days artemisinin-based combination therapy (ACT) plus 0.75 mg/body weight PQ at day 1; *P. vivax* is treated with 3 days ACT plus 14 days PQ 0.25 mg/body weight; and, *P. malariae* infection is treated for 3 days ACT only without PQ. Given the uncertainty in parasite identification and the possibility of mixed infection, all patients were treated as if positive for both *P. falciparum* and *P. vivax.* These issues highlight the need to include *P. knowlesi* in malaria microscopy diagnosis training, but also to insert treatment for *P. knowlesi* infection in the national malaria treatment guidelines. According to the WHO treatment guidelines [20] and a review of *P. knowlesi* treatment by Barber et al. [21], administration of ACT for uncomplicated *P. knowlesi* is recommended in areas where resistance to chloroquine (CQ) is common. Chloroquine resistance to *P. falciparum* was first reported in Indonesia in 1983 [22], in the 1990s for *P. vivax* [23, 24], and in 2000 for *P. malariae* [25]. Therefore, using ACT for *P. knowlesi* cases in Indonesia is consistent with WHO’s treatment guidelines.

However, PQ is not appropriate for *P. knowlesi* treatment for several reasons. As *P. knowlesi* does not have a hypnozoite form, and rapid asexual and sexual stage clearance occurs without utilising PQ, use of the drug provides no clear benefit [21]. Moreover, as point of care testing for glucose-6-phosphate dehydrogenase deficiency is not yet done in Indonesia, there is some risk in administration of the drug [2]. In cases where PCR detects *P. vivax* which microscopy has misdiagnosed as *P. knowlesi*, then PQ treatment is indicated [21]. In this report, the MHO Sabang gave PQ to all patients because of inconsistent microscopy results and late receipt of PCR diagnosis approximately 2 weeks to one month after the case had been treated. Delayed receipt of PCR examination results highlights the need to establish national or regional laboratory centres for *P. knowlesi* diagnosis. Furthermore, in areas like Sabang where human malaria has been eliminated, all malaria cases diagnosed by microscopy must be re-examined by PCR [3]. WHO recommends to standardization of PCR protocols for *P. knowlesi* diagnosis and with appropriate quality assurance protocols in place [26].

*Plasmodium knowlesi* can cause severe disease with a high case fatality rate [27–29]; in Sabah, Malaysia the parasite caused severe malaria in 29% of 130 infections [27]. In Sabah, severe manifestations included parasitemia > 100,000/μL, jaundice, respiratory distress/pulmonary oedema, hypotension, hypoglycaemia, lactic acidosis, and acute kidney injury [27, 29]. In our findings from Sabang, three cases could be classified as severe because of hemoglobinuria. All cases were treated with oral ACT + PQ as they could follow commands despite a Glasgow Coma Score of 12–13. Of the three severe cases, one case had parasitemia 24,368/μL, and another case had parasite density of 14,620/μL. Interestingly, one case with hemoglobinuria had low parasitemia, 2300/μL. Of three cases with unconsciousness, one case had low parasite density, 48/μL. Additional laboratory findings are not available, as most patients were treated in primary health centres with limited facilities.

Although Sabang successfully interrupted transmission of human plasmodium species, its experience with *P. knowlesi* highlights the need for western Indonesia and other countries in southeast Asia to incorporate strategies for surveillance, treatment, and control of this zoonotic parasite. Consistent with WHO recommendations, the National Malaria Control Program (NMCP) of Indonesia has included *P. knowlesi* in its formal reporting system beginning in 2018, and discussion as to how best to set up appropriate surveillance in ongoing. The NMCP has also begun collaboration with the Ministry of Forestry and its Nature Conservation Unit on possible control strategies for macaques [communication with dr Elvieda, NMCP manager].

## Abbreviations

ACT: artemisinin-based combined therapy
CI: case investigation
DHP: dihydroartemisin piperaquine
DOT: direct observation treatment
IRS: indoor residual spraying
LLIN: long-lasting insecticide-treated bed nets
MHO: municipal health office
PCR: polymerase chain reaction
PHC: primary health centre
PQ: primaquine
QA: quality assurance
RACD: reactive case detection
WHO: World Health Organization
